# The impact of deep versus standard neuromuscular block on intraoperative safety during laparoscopic surgery: an international multicenter randomized controlled double-blind strategy trial — EURO-RELAX TRIAL

**DOI:** 10.1186/s13063-021-05638-2

**Published:** 2021-10-26

**Authors:** Maarten Honing, Gabby Reijnders-Boerboom, Salome Dell-Kuster, Monique van Velzen, Chris Martini, Franco Valenza, Paolo Proto, Oscar Díaz Cambronero, Suzanne Broens, Ivo Panhuizen, Margot Roozekrans, Thomas Fuchs-Buder, Martijn Boon, Albert Dahan, Michiel Warlé

**Affiliations:** 1 Leiden University Medical Center, Leiden, The Netherlands; 2 Netherlands Cancer Institute, Amsterdam, The Netherlands; 3 Radboud University Medical Center, Nijmegen, The Netherlands; 4 Canisius Wilhelmina Ziekenhuis, Nijmegen, The Netherlands; 5 University and University Hospital Basel, Basel, Switzerland; 6 Istituto Nazionale Dei Tumori, Milano, Italy; 7 Hospital Universitari I Politecnic La Fe, Valencia, Spain; 8 Noordwest Ziekenhuisgroep, Alkmaar, The Netherlands; 9 Université De Lorraine, Nancy, France

**Keywords:** ClassIntra® grade, Deep neuromuscular block, Monitoring, Neuromuscular, Surgical working conditions

## Abstract

**Background:**

Muscle relaxants are routinely used during anesthesia to facilitate endotracheal intubation and to optimize surgical conditions. However, controversy remains about the required depth of neuromuscular block (NMB) needed for optimal surgical working conditions and how this relates to other outcomes. For instance, a deep neuromuscular block yields superior surgical working conditions compared to a standard NMB in laparoscopic surgery, however, a robust association to other (safety) outcomes has not yet been established.

**Methods:**

Trial design: an international multicenter randomized controlled double-blind strategy trial.

Trial population: 922 patients planned for elective, laparoscopic or robotic, abdominal surgery.

Intervention: Patients will be randomized to a deep NMB (post-tetanic count 1–2 twitches) or standard care (single-dose muscle relaxant administration at induction and repeated only if warranted by surgical team).

Main trial endpoints: Primary endpoint is the difference in incidence of intraoperative adverse events during laparoscopic surgery graded according to ClassIntra® classification (i.e., ClassIntra® grade ≥ 2) between both groups. Secondary endpoints include the surgical working conditions, 30-day postoperative complications, and patients’ quality of recovery.

**Discussion:**

This trial was designed to analyze the effect of deep neuromuscular block compared to standard neuromuscular block on intra- and postoperative adverse events in patients undergoing laparoscopic surgery.

**Trial registration:**

ClinicalTrials.gov NCT04124757(EURO-RELAX); registration URL: https://clinicaltrials.gov/ct2/show/NCT04124757, registered on October 11th, 2019.

## Administrative information

**Table:** 

Title {1}	*The impact of deep versus standard neuromuscular block on intra-operative safety during laparoscopic surgery: an international multicenter randomized controlled double-blind strategy trial – EURO-RELAX TRIAL*
Trial registration {2a and 2b}.	ClinicalTrials.gov identifier NCT04124757 (EURO-RELAX) Registered on October 11, 2019
Protocol version {3}	Version 1.4; February 2020
Funding {4}	Trial Sponsor: Leiden University Medical Center Collaborator: Merck Sharp & Dohme corp., Kenilworth U.S.A.
Author details {5a}	Maarten Honing^1,6^; Gabby Reijnders-Boerboom^2,7^; Salome Dell-Kuster^3^; Monique van Velzen^1^; Chris Martini^1^; Franco Valenza^4^; Paolo Proto^4^; Oscar Díaz Cambronero^5^; Susanne Broens^6^; Ivo Panhuizen^7^; Margot Roozekrans^8^; Thomas Fuchs-Buder^9^; Martijn Boon^1^; Albert Dahan^1^; Michiel Warlé^2^ 1. Leiden University Medical Center, Leiden, The Netherlands 2. Radboud University Medical Center, Nijmegen, The Netherlands 3. University and University Hospital Basel, Basel, Switzerland 4. Istituto Nazionale Dei Tumori, Milano, Italy 5. Hospital Universitari I Politecnic La Fe, Valencia, Spain 6. Netherlands Cancer Institute, Amsterdam, The Netherlands 7. Canisius Wilhelmina Ziekenhuis, Nijmegen, The Netherlands 8. Noordwest Ziekenhuisgroep Alkmaar, The Netherlands 9. Université De Lorraine, Nancy, France
Name and contact information for the trial sponsor {5b}	Albert Dahan, Leiden University Medical Center, Leiden, The Netherlands; a.dahan@lumc.nl
Role of sponsor {5c}	The principal investigator is the trial sponsor; responsible for trial design, execution, analysis and publication of outcomes. The funding parties did not influence trial design, nor will they influence data collection, data analysis, data interpretation or manuscript writing.

## Introduction

### Background and rationale {6a}

Muscle relaxants are routinely given during general anesthesia to facilitate endotracheal intubation and to improve surgical exposure. Traditionally, muscle relaxants were not given in high doses due to the risk of residual neuromuscular block at the end of the procedure. However, novel reversal techniques have now made a deep neuromuscular block feasible in clinical practice. The application of a deep neuromuscular block (NMB) over a standard NMB may have several advantages for the surgical team and the patient. Primarily, deep NMB provides a superior motionless surgical field, resulting in optimal surgical working conditions. This was shown by multiple independent studies for a variety of laparoscopic surgeries [1–8]. These findings are important since laparoscopic (robotic) surgery is continuously evolving and increasingly entails complex procedures where space is limited. These complex procedures require optimal surgical working conditions for the surgeon to perform the surgery effectively and safely. Provision of a motionless surgical field may also reduce the incidence of intraoperative adverse events. Indeed, the incidence of sudden movements during a surgical procedure is significantly reduced when a deep NMB was applied. In addition, a motionless surgical field may also reduce the amount of excess tissue damage that is inflicted during the execution of the procedure. And finally, muscle relaxants may have pleiotropic effects on immune cells that express nicotinic acetylcholine receptors [9], resulting in favorable perioperative immune homeostasis. Combined, these effects make it plausible that a deep NMB improves postoperative outcomes beyond the beneficial intraoperative effects that have been noted before. A previous retrospective study found that deep NMB was associated with fewer postoperative infections and a reduced rate of unplanned readmissions within 30 days after surgery [10]. However, there are currently no large prospective trials to corroborate or refute any effects of deep NMB on perioperative outcomes. The current trial was designed to fill this gap in evidence. The EURO-RELAX trial is an international multicenter randomized controlled double-blind strategy trial that studies the effect of a deep NMB (PTC 1–2 twitches), in comparison to standard NMB (single induction dose rocuronium), on the incidence of intraoperative adverse events and postoperative outcomes in a variety of laparoscopic surgical procedures.

### Objectives {7}

#### Primary endpoint

To study the incidence of intraoperative adverse events of grade ≥ 2 according to ClassIntra® (classification of intraoperative adverse events [11], Table 1) in patients undergoing laparoscopic or robotic abdominal surgeries of higher complexity during deep neuromuscular block versus standard neuromuscular block [11].

**Table 1:** Definitions of ClassIntra® classification for intraoperative adverse events [14]

Grade 0	No deviation from the ideal intraoperative course
**Grade 1**	Any deviation from the ideal intraoperative course: • Without the need for any additional treatment or intervention • Patient asymptomatic or mild symptoms
**Grade 2**	Any deviation from the ideal intraoperative course : • With the need for any additional minor treatment or intervention • Patient with moderate symptoms, not life- threatening and not leading to permanent disability
**Grade 3**	Any deviation from the ideal intraoperative course: • With the need for any additional moderate treatment or intervention • Patient with severe symptoms, potentially life- threatening and/or potentially leading to permanent disability
**Grade 4**	Any deviation from the ideal intraoperative course: • with the need for any additional major treatment or intervention • patient with life-threatening symptoms and/or leading to permanent disability
**Grade 5**	Any deviation from the ideal intraoperative course with intraoperative death of the patient

The classification defines intraoperative adverse events as any deviation from the ideal intraoperative course occurring between skin incision and skin closure*.* Any event related to surgery and anesthesia during the index surgery must be considered and should be rated directly after surgery. The following events are not defined as intraoperative complications: sequelae, failures of cure, events related to the underlying disease, wrong-site or wrong-patient surgery, or errors in indication

#### Secondary endpoints

To assess the effect of deep neuromuscular block compared to standard neuromuscular block on the following:

1. Surgical working conditions (Leiden Surgical Rating Scale; L-SRS)
2. Patients’ early quality of recovery (QoR-40 and SF-36) [12–14]
3. 30-day postoperative complications (Clavien-Dindo classification and Comprehensive Complication Index (CCI)) [15, 16]
4. Unplanned hospital readmission rate after laparoscopic surgeries

### Trial design {8}

The EURO-RELAX is an international multicenter randomized controlled double-blind strategy trial. Patients scheduled for elective laparoscopic and robotic surgery of higher complexity will be randomized to receive a deep or standard neuromuscular block.

## Methods: Participants, interventions, and outcomes

### Study setting {9}

**Fig. 1:**
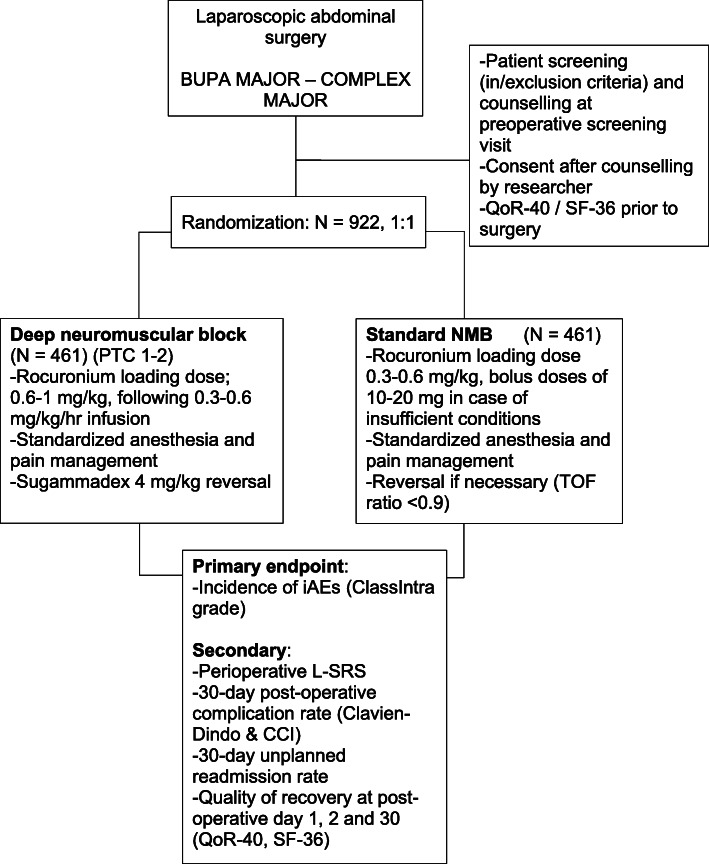
Study procedures flow diagram. CCI, Comprehensive Complication Index; L-SRS, Leiden Surgical Rating Scale; NMB, neuromuscular block; TOF, train-of-four; QoR-40, Quality of Recovery; SF-36, Short-Form 36

The trial will be performed in academic and non-academic public hospitals in France, Italy, the Netherlands, and Spain; a list of participating centers is enclosed in the Appendix. An overview of trial procedures is represented in Fig. 1 and Table 2.

**Table 2:** Schematic schedule of enrolment, interventions, and study outcomes

		Study period					
	Enrolment	Allocation	Surgery	Follow-up			Close-out
***Timeframe***	***− 14 days***	***OR day***	***OR day***	***+ 1 day***	***+ 2 days***	***+ 3 days***	***+ 30 days***
Eligibility screening	X						
Informed consent	X						
Randomization		X					
**Intervention**							
Standard or deep NMB			X				
**Primary, secondary, and postoperative outcomes**							
ClassIntra Grade® [14]			X				
L-SRS [6, 23]			X				
QoR-40 [16, 17]	X			X	X		
SF-36 [15]	X						X
Clavien-Dindo and CCI [18, 19]				X	X	X	X
Readmission rate							X
NRS and administered analgesics	X		X	X	X	X	

*CCI* Comprehensive Complication Index, *L-SRS* Leiden Surgical Rating Scale, *NMB* neuromuscular block, *NRS* Numeric Rating Scale, *QoR-40* Quality of recovery, *SF-36* Short-Form 36

### Eligibility criteria {10}

In order to be eligible for trial participation, a patient must meet all of the following inclusion criteria:

▪ Scheduled for elective laparoscopic or robotic abdominal procedures (see Table 3 for an overview of examples of eligible procedures) with a complexity of the surgical procedure of “MAJOR”, “MAJOR PLUS”, or “COMPLEX MAJOR” according to the BUPA classification [18, 19].

**Table 3:** Examples of BUPA classification [20, 21] for case complexity of eligible procedures

• BUPA MAJOR	- Cholecystectomy
• BUPA MAJOR PLUS	- Colorectal resection - Nephrectomy - Hysterectomy - Adrenalectomy (unilateral) - Right colectomy - Partial nephrectomy - Gastric sleeve - Gastric bypass - Donor nephrectomy - Left colectomy - Sigmoidectomy - Partial stomach resection
• BUPA COMPLEX MAJOR	- Low anterior resection - Partial hepatectomy - Prostatectomy - Hemi hepatectomy - Esophagostomy - Pyeloplasty - Stomach resection

▪ ASA Physical Status class I-III

▪ ≥ 18 years of age

▪ Able to give oral and written informed consent

The following exclusion criteria are being applied:

▪ Low or intermediate complexity laparoscopic procedures (BUPA “SIMPLE” or “INTERMEDIATE”) [18, 19]

▪ Known or suspected neuromuscular disorders impairing neuromuscular function

▪ Allergies to muscle relaxants, anesthetics, or narcotics mentioned in the trial methods

▪ A (family) history of malignant hyperthermia

▪ Women who are or may be pregnant or are currently breast-feeding

▪ Chronic use of any type of opioid or psychotropic drug

▪ Chronic use of NSAID for treatment of chronic pain

▪ Indication for rapid sequence induction

▪ Contra-indication for sugammadex use (e.g., known sugammadex allergy or GFR < 30 ml/min)

### Informed consent procedures {26a}

Patients will receive general trial information by an anesthesia caregiver at routine preoperative screening visit. All possible trial candidates will receive a copy of the patient information sheet and consent form. If the patient is willing to participate, oral and written consent will be obtained in person by the local investigator or study nurse after initial screening.

### Additional consent provisions for collection and use of participant data and biological specimens {26b}

Not applicable.

## Interventions

### Explanation for the choice of comparators {6b}

The comparator group will receive a standard neuromuscular block which resembles current practice in the majority of hospitals. A standard neuromuscular block entails the administration of a single bolus dose of the muscle relaxant rocuronium at a dose of 0.3–0.6 mg/kg at the induction of anesthesia. In case of a residual neuromuscular block at the end of anesthesia (i.e., a Train-of-four (TOF) ratio < 0.9), sugammadex at a dose of 2.0 mg/kg will be used for reversal.

### Intervention description {11a}

The intervention group will receive a deep neuromuscular block throughout the surgical procedure. A deep neuromuscular block is established by administration of a bolus rocuronium at a dose of 0.6–1.0 mg/kg at the induction of anesthesia. Thereafter, a continuous infusion of rocuronium (0.3–1.0 mg/kg/h) is started and titrated to maintain a neuromuscular block of 1–2 twitches post-tetanic count (PTCs). PTCs will be measured by 5-min intervals, when required continuous rocuronium infusion is adjusted at 15-min intervals by steps of 0.1–0.2 mg/kg/h. The neuromuscular block will be reversed with sugammadex at a dose of 4.0 mg/kg at the end of surgery.

### Criteria for discontinuing or modifying allocated interventions {11b}

In case of suboptimal surgical working conditions (L-SRS ≤ 3) or an intraoperative adverse event (ClassIntra® grade ≥ 2) [11], the following measures may be taken (and will be recorded):

▪ Administration of rocuronium, 10–20 mg (repeated on request)

▪ Administration of propofol, 20–50 mg

▪ Additional bolus of opioids (remifentanil, sufentanil, fentanyl)

▪ Increase of intra-abdominal pressure (IAP) (12 mmHg)

If subsequent administration of rocuronium, opioids, or analgesics or an increase of intra-abdominal pressure remains insufficient, conversion to a deep NMB may be instituted.

#### Patient drop-out after withdrawal of consent

When the patient withdraws consent prior to surgery he or she will not be treated according to trial protocol and will be replaced by another patient. Patients who withdraw consent post-procedure will immediately be excluded from the trial and are not replaced. Data acquired prior to the patient’s withdrawal will be included in final data analysis.

### Strategies to improve adherence to interventions {11c}

Obtaining and maintaining deep or standard NMB, as per-protocol, will be supervised by a local researcher with ample experience in the field of anesthesia and administration of neuromuscular blocking agents. He or she is not involved in scoring of the primary outcome.

### Relevant concomitant care permitted or prohibited during the trial {11d}

There are no restrictions regarding concomitant care. Apart from the depth of neuromuscular block, anesthesia and surgery will follow standard procedures. General trial procedures are outlined below.

#### Intraoperative anesthesia procedures

Standard anesthesia intravenous access and standard monitoring according to local institutional protocol are applied. General anesthesia will be induced with propofol and maintained with propofol, sevoflurane or desflurane. Intraoperative antinociceptive treatment will be with sufentanil, fentanyl, or remifentanil. The choice of hypnotic and opioid is upon the discretion of the attending anesthesiologist. Hypnotic depth will be routinely monitored with bispectral index (BIS; Philips, Amsterdam, the Netherlands) or entropy monitoring (Entropy Module, GE Healthcare, Helsinki, Finland). The target level of the BIS is 50 ± 5 during the procedure, whilst the entropy target is 40 ± 5 to avoid under- or overdosing of the hypnotic agent. End-tidal pCO_2_ will be maintained at 4.5 to 5.5 kPa, and central body temperature will be kept between 36 and 37° C by using forced warm air blankets.

All participating patients will receive neuromuscular monitoring according to international guidelines for neuromuscular monitoring in research [20]. Neuromuscular monitoring will exclusively be applied at the *m. adductor pollicis* of one of the free moving thumbs with either TOF-scan (Draeger Medical; Hemel Hempstead, UK), TOF-Watch (MSD, Haarlem, The Netherlands), or GE-electromyography (General Electric, Helsinki, Finland). All monitor types can be used interchangeably in practice of this trial. All monitors will be applied in accordance with the guidelines of the manufacturer, including any baseline and calibration procedures; these will take place after the patient has been put under general anesthesia, but before the administration of any neuromuscular blocking agent. Baseline TOF ratio will be noted in the case report form (CRF). Patients may only be extubated when the corrected TOF ratio is at least 0.9 [21].

#### Intraoperative surgical procedures

All laparoscopic or robotic procedures will exclusively be performed at standard intra-abdominal insufflation pressures. After insufflation, intra-abdominal volume will be recorded as indirect measure of abdominal wall compliance and surgical workspace.

All surgeons and anesthesiologists will be trained prior to start of the trial in the use of ClassIntra® [11] and L-SRS grades to ensure consistent scoring between the centers. Training of involved and anesthesiologists and will be done by a group of dedicated surgeons, anesthesiologists, and researchers that form a blinded adjudication committee (BAC). The BAC can review each case based on the surgical report. The first two cases in each center will be evaluated by the BAC. Furthermore, evaluation of consistent reporting of the primary outcome will be performed by the BAC after 10, 20, 50, and 100 cases to check for inconsistencies.

#### Postoperative procedures

The patient will be followed up at the (recovery) ward. Pain relief at the post-anesthetic care unit (PACU) and ward is left at discretion of the attending anesthesiologist.

### Provisions for post-trial care {30}

Postoperative complications will be treated as indicated by the surgeon or anesthesiologist. No specific post-trial provisions apply.

### Outcomes {12}

#### Main trial parameter/endpoint

The incidence of symptomatic intraoperative adverse events requiring intervention or treatment (ClassIntra®grade ≥ 2 [11], Table 1) during laparoscopic surgery in the standard of care versus the deep NMB group, as scored by the attending surgeon and anesthesiologist at the end of every procedure

#### Secondary trial parameters/endpoints

▪ The intraoperative surgical conditions (L-SRS; 5-point scale scored at 15-min intervals) [6, 17]

▪ Quality of recovery based on the validated QoR-40 [13] questionnaire on postoperative day 1 and 2

▪ Quality of life based on the validated SF-36 [12] questionnaire on postoperative day 30

▪ Incidence of 30-day postoperative complications according to the Clavien-Dindo classification and Comprehensive Complication Index (CCI) [15]. The Clavien-Dindo classification categorizes complications from mild to serious (grade I any deviation to grade V death) [16].

▪ 30-day unplanned hospital readmission rate

### Participant timeline {13}

A schedule of trial enrolment, interventions and outcomes is enclosed in Table 2.

### Sample size {14}

Preliminary data of the ClassIntra® validation trial indicated that the incidence of intraoperative ClassIntra® grade ≥ 2 (Table 1) [11] is approximately 20% for BUPA major-complex major procedures. We evaluated the effect size of deep NMB on perioperative complication rate in pooled data of four prospective studies that evaluated the effect of deep NMB on surgical conditions [22–25]. Complication rate dropped from 13% in the standard-of-care group to 5% in the deep NMB group. Based on this, we consider a 40% relative reduction of symptomatic intraoperative adverse events requiring intervention or treatment (ClassIntra® grade ≥ 2) a realistic and clinically relevant outcome for this study.

With an alpha of 5% and beta of 90%, 439 patients are required in each arm of the trial (in total 878 patients; G*Power statistics 3.1.9.7; HHU; Dusseldorf; Germany [26]). In accordance with our experience in previous trials, we expect a 5% drop-out rate. Therefore, a total of 922 patients should be randomized 1:1 in this trial. Reasons for patient drop-out are as follows:

- Conversion to open surgery within the first 20 min of surgery due to unforeseen adhesions, tumor progression, (peritoneal) metastases, or other diagnosis
- Pre-incision alteration of surgical plan to laparotomy instead of laparoscopy after patient randomization to deep NMB or standard-of-care group
- Patient safety concerns at induction of anesthesia, e.g., anaphylaxis at induction of anesthesia or unanticipated difficult airway

### Recruitment {15}

Patients will be screened for eligibility during the routine preoperative visit at the anesthesia outpatient clinic. Potential candidates will receive oral and written general trial information by an anesthesia caregiver. Several days after the preoperative visit, a research employee will contact the patient to further inform the patient about the trial procedures and to obtain informed consent.

## Assignment of interventions: allocation

### Sequence generation {16a}

Patient will be randomized to group 1 (deep NMB) or group 2 (standard NMB) prior to surgery by an independent researcher. The randomization sequence is generated by a dedicated computer randomization software, Castor (Castor EDC, CIWIT B.V., www.castoredc.com), and is stratified by center and by BUPA category (MAJOR, MAJOR PLUS or COMPLEX MAJOR, a list of examples is provided in Table 3) [18, 19]. To ensure a balanced distribution among randomization arms, randomization will be performed using randomly varying block sizes of two and four.

### Concealment mechanism {16b}

Central randomization is provided through an online randomizer to ensure allocation concealment.

### Implementation {16c}

During every procedure an unblinded researcher will be present to ensure maintenance of the desired level of NMB and trial protocol adherence.

## Assignment of interventions: blinding

### Who will be blinded {17a}

Participating patients and the surgical and anesthesia teams, as well as postoperative caregivers, will be blinded for treatment allocation. During every procedure, an unblinded researcher will ensure adherence to the trial protocol.

To avoid unblinding of the surgical and anesthesia teams, a syringe pump with rocuronium will be prepared for every patient, regardless of the treatment allocation. The rocuronium syringe and neuromuscular monitor will be covered in such way that the medical teams in the operating room will not be able to read infusion rates or depth of NMB.

ClassIntra® grade scoring will be done by the blinded surgeon and anesthesiologist; the unblinded researcher will not be involved in the ClassIntra® grade scoring. The investigators who assess postoperative secondary endpoints or perform final data analysis are blinded to group allocation.

Participating patients remain blinded until study completion.

### Procedure for unblinding if needed {17b}

After the ClassIntra® grade has been obtained by both the surgeon and anesthesiologist, the anesthesiologist will be unblinded regarding the depth of NMB to ensure adequate NMB reversal.

As described in paragraph {11b}, the unblinded researcher may administer additional rocuronium if the surgery requires administration of additional muscle relaxants. These procedures assure that unblinding during the procedure is not needed.

Only when the attending anesthesiologist deems it necessary for patient safety to take over neuromuscular management may this lead to unblinding of the anesthesiologist only. The surgeon remains blinded in this occasion. Unblinding will be noted on the CRF and reported in the final manuscript.

## Data collection and management

### Plans for assessment and collection of outcomes {18a}

After informed consent and prior to randomization, the following patient baseline data will be acquired:

▪ Age, sex, length (m), and weight (kg)

▪ Heart rate (/min) and blood pressure (mmHg)

▪ Planned procedure and center of admission

▪ ASA Physical Status Class and concurrent comorbidities

▪ Baseline NRS (Numeric Rating Scale; 0 no pain to 10 worst pain imaginable)

▪ QoR-40 [13, 14] and SF-36 [12] questionnaires

#### Intraoperative data collection

During the procedure, the following variables are collected, at 15-min intervals after abdominal insufflation, until the end of the surgery:

▪ Leiden surgical rating scale (L-SRS) [6, 17]

▪ Heart rate (/min), blood pressure (mmHg)

▪ Intra-abdominal pressure (mmHg) and total insufflation volume (liters)

▪ Depth of anesthesia: Bispectral index or entropy, and end-tidal inhalational an anesthetic concentration (if applicable)

▪ Depth of NMB (TOF count/ratio and/or PTC)

At the end of the case the following variables will be collected:

▪ ClassIntra® grade [11]

▪ Duration of surgery and anesthesia (minutes)

▪ When applicable, reason of conversion to open procedure

▪ Time of extubation

▪ Cumulative drug dosages (propofol, opioids, muscle relaxant, reversal agent, inotropes, NSAIDs, or metamizole)

▪ Core temperature in degrees Celsius

▪ Surgical satisfaction with the anesthesia: “very dissatisfied”, “dissatisfied”, “satisfied”, to “very satisfied”

#### Postoperative recovery data at the PACU at 15-min intervals

▪ Discharge readiness (Aldrete score ≥ 9 and NRS < 5)

▪ Heart rate (/min) and blood pressure (mmHg)

▪ Peripheral oxygen saturation, respiratory rate (/min), and supplemental O_2_ administration (liters/minute)

▪ Pain level (NRS) and administration of analgesics and anti-emetics

▪ Ramsay sedation scale [27, 28] and Aldrete score [29]

▪ Nausea or vomiting

#### Postoperative recovery data until postoperative day 30

▪ Length of PACU and hospital admission and, if applicable, length of ICU admission

▪ NRS, at movement, three times daily until hospital discharge or to a maximum of 72 h

▪ Daily administered analgesics and anti-emetics until hospital discharge or to a maximum of 72 h

▪ QoR-40 quality of recovery score [13, 14], filled in by the blinded patient at day 1 and 2 after surgery

▪ SF-36 quality of recovery score [12], filled in by the blinded patient at day 30 after surgery

▪ 30-day unplanned hospital readmission rate, reported by the blinded patient and evaluated by a blinded researcher based on clinical charts

▪ Day 30 complication rate, by a blinded researcher, local researcher, or research nurse who assess clinical discharge letters and clinical charts at postoperative day 30, based on the Clavien-Dindo scale and comprehensive complication index [15, 16]

### Plans to promote participant retention and complete follow-up {18b}

The trial timeline (Table 2) depicts that most data will be collected during clinical observation, where the researcher is present. On postoperative day 1 and 2, the researcher will visit the patient on the ward to ensure adequate postoperative follow-up. Patients will be contacted by the local researcher incase questionnaires have not yet been completed.

### Data management {19}

A local subject pseudonymization key file will be stored at each individual site; data confidentiality will be ensured by measures described in paragraph {27}. For trial data capturing, we will use electronic data capture (EDC) via eCRFs (Castor EDC, CIWIT B.V., www.castoredc.com) specifically designed to capture trial information with an audit log trail and enabling data entry by both the researcher and patient through the eCRF system. To minimize data entry errors, data will be range checked during data entry; a stop error will occur when entered data is outside the expected range. Intraoperative data at the operation theater, recovery room, and ward will be generated by direct data entry by the local researcher. Source data will be stored at the specific trial site where it originated and will be safeguarded by the local researcher.

Only authorized investigators and personnel can correct or complete eCRFs, and all corrections will be documented in an audit log trail. All data will be handled confidentially and in a pseudonymized fashion. After the database has been declared complete and accurate, the EDC database will be locked. The sponsor, local researchers, and project leader are responsible for data processing. If a subject withdraws consent, data collected until that moment will be used. All data will be stored for the length of the trial and for 15 years afterwards, for further analyses and publication. All handling of personal data will comply with the Dutch Personal Data Protection Act. A data management plan is available on file with additional details regarding data storage and handling.

### Confidentiality {27}

All patients will receive a random subject identification code. Patient identifying data will be omitted. The encrypted codebook will be stored digitally and will be safeguarded by the local investigator. Source data will be stored at the specific trial site where it originated and will be safeguarded by the local investigator. Data sent to the investigator will only contain this code and will not contain identifying data. Other involved parties (data monitoring committee, Health and Youth Care Inspectorate) could be granted access to patient data, also patient identifying data, to review if the research is being executed safely. These involved parties will handle the patient identifying data in a confidential manner.

### Plans for collection, laboratory evaluation, and storage of biological specimens for genetic or molecular analysis in this trial/future use {33}

Not applicable.

## Statistical methods

### Statistical methods for primary and secondary outcomes {20a}

## Primary study parameter

The incidence of adverse events (ClassIntra® grade ≥ 2) between randomization groups will be compared using a Chi-squared test; *p* < 0.05 will be considered significant. Data will be analyzed according to the intention-to-treat principle.

## Secondary study parameters

Continuous normally distributed variables will be expressed as means and standard deviations or when not normally distributed as medians and interquartile ranges. Categorical variables will be expressed as absolute and relative frequencies. To statistically compare groups, Student’s *t* tests will be used, if continuous data are not normally distributed, the Mann-Whitney *U* test will be used. Categorical variables will be compared using a Chi-squared test or Fisher’s exact tests. *P*-values < 0.01 will be considered significant for secondary outcome variables. Data analyses will be performed using R Studio, Boston, MA, USA.

Longitudinal data (heart rate, blood pressure, L-SRS, BIS, per depth of NMB at the given moment) will be analyzed using linear mixed models using NONMEM (ICON Development Solutions, Ellicott, MD, USA).

### Interim analyses {21b}

No interim analysis will be performed.

### Methods for additional analyses (e.g., subgroup analyses) {20b}

In subgroup analyses, the incidence ClassIntra® grade ≥ 2 will be analyzed per BUPA category and type of anesthetic (total intravenous versus volatile anesthesia). Demographic data, intraoperative administered cumulative drugs dosages, postoperative NRS scores, and length of admission (at PACU, ICU, and/or ward) will be reported. Data analyses will be performed as described in the paragraph secondary trial parameters {20a}.

### Methods in analysis to handle protocol non-adherence and any statistical methods to handle missing data {20c}

Data will be analyzed according to the intention-to-treat principle for the primary outcome. Per-protocol analyses will be performed as sensitivity analysis. Data consistency is checked at data entry and prior to data analyses, via range checks and by visual evaluation of data distribution in a random sample of patients. Missing data of trial outcome measures and missing independent variables will be imputed by multiple imputation if the percentage of missing data in important variables exceeds 10%.

### Plans to give access to the full protocol, participant level-data, and statistical code {31c}

The trial protocol and statistical analysis plan are available upon request. Anonymized trial data and statistical codes are available upon request from the corresponding author.

## Oversight and monitoring

### Composition of the coordinating center and trial steering committee {5d}

LUMC and Radboud UMC are the coordinating centers. The trial steering committee (TSC) is composed of Monique van Velzen, Maarten Honing, Albert Dahan, Chris Martini, Martijn Boon, Michiel Warlé, and Gabby Reijnders-Boerboom. The TSC will oversee whether data collection and intraoperative procedures are in accordance with the trial protocol.

In addition, an adjudication committee (BAC), consisting of members of the STC and local investigators of actively recruiting centers, will discuss on a regular basis progress of the trial and review every adverse event of classic grade 2 or higher, or any uncertainties in scoring of the primary outcome. This is to ensure uniform and consequent scoring of the primary outcome among centers. The BAC will be blinded to the treatment allocation. Minutes of the BAC will be made and stored by members of the TSC.

### Composition of the data monitoring committee, its role, and reporting structure {21a}

The on-site monitoring will consist of the data monitoring committee (DMC), which will control presence and completeness of the research files and informed consent forms. Source data checks will be performed as described in the monitoring plan. Every participating center will be visited and monitored at least once every year. All trial sites will be monitored by a trained, unblinded, independent monitor. The DMC will report directly to the principal investigator and the monitored local center. Additional monitoring details are specified in a dedicated monitoring plan.

### Adverse event reporting and harms {22}

Adverse events (AEs) are defined as any undesirable experience occurring to a subject during the trial, whether considered related to the trial procedure.

A serious adverse event (SAE) is any untoward medical occurrence or effect:

- Results in death
- Is life threatening (at the time of the event)
- Requires hospitalization or prolongation of existing inpatients’ hospitalization
- Results in persistent or significant disability or incapacity
- Is a congenital anomaly or birth defect
- Any other important medical event that did not result in any of the outcomes listed above due to medical or surgical intervention but could have been based upon appropriate judgment by the investigator. An elective hospital admission will not be considered as a serious adverse event.

All (S)AEs reported spontaneously by the subject or observed by the investigator or his staff will be recorded, only if judged to be substantial deviating from expected standard clinical course. This includes SAEs that influence postoperative recovery or clinical outcome.

The investigator will report all SAEs to the sponsor without undue delay after obtaining knowledge of the events. Subjects will be followed up for AEs and SAEs until the final trial procedures or 7 days after discontinuation of the trial. All reports will be digitally filed in the electronic clinical data capture form.

The sponsor will report the SAEs to the accredited Medical Research and Ethics Committee (MREC) Leiden-Den-Haag-Delft, within 7 days of first knowledge for SAEs that result in death or are life threatening followed by a period of maximum of 8 days to complete the initial preliminary report. All other SAEs will be reported within a period of maximum 15 days after the sponsor has first knowledge of the serious adverse events.

### Frequency and plans for auditing trial conduct {23}

Any trials conducted by the sponsor may be audited at random by an independent auditing committee. No preplanned auditing is scheduled.

### Protocol amendments

A “substantial amendment” is defined as an amendment to the terms of the MREC application or to the protocol or any other supporting documentation that is likely to affect to a significant degree:

- The safety or physical or mental integrity of the subjects of the trial
- The scientific value of the trial
- The conduct or management of the trial
- The quality or safety of any intervention used in the trial

Any protocol amendment will not be implemented prior to MREC approval.

### Plans for communicating important protocol amendments to relevant parties (e.g., trial participants, ethical committees) {25}

All substantial amendments will be notified to the MREC and to the competent authority.

Following MREC approval, the amendments will be communicated immediately to all local investigators of participating centers via email.

### Dissemination plans {31a}

The trial protocol and analysis plan are registered at ClinicalTrials.gov (NCT04124757 (EURO-RELAX)). The results of the trial will be published in (inter-)national scientific journals and at international congresses in the field of anesthesiology and/or surgery. The results of this trial will be disclosed unreservedly, including to trial participants, according to the Central Committee on Research Involving Human Subjects (CCMO) statement on publication policy. Material for public dissemination will be submitted to the MREC for review prior to submission for publication.

### Discussion

Muscle relaxants are commonly given to optimize surgical working conditions during surgical procedures requiring general anesthesia. Application of a deep neuromuscular block (i.e., a post-tetanic count of 1–2 twitches) over a standard neuromuscular block (single shot induction dose of muscle relaxant) improves surgical exposure during laparoscopic surgery by fully relaxing abdominal and diaphragmic muscles and by preventing any sudden unexpected movement of the patient during a procedure [1–8]. However, whether intraoperative beneficial effects of deep NMB translate to better perioperative outcomes is currently not well investigated. There are however a few hypothesized mechanisms, by which a deep NMB may improve outcomes. First, it is plausible that a perfectly motionless surgical field improves tissue handling by the surgeon and mitigates tissue damage inflicted during the execution of the procedure. In addition, deep NMB may improve intraoperative safety by fully preventing any unexpected gross movement (i.e., bucking, coughing, or contraction of abdominal muscles) to occur, which could result in surgical instruments to inflict harm to intra-abdominal organs of the patient. Finally, various studies have noted beneficial immune effects of muscle relaxants [9, 30]. Muscle relaxants block various (sub-)types of the nicotinic acetylcholine receptor. These subtypes are expressed on macrophages and are involved in the nicotinic anti-inflammatory pathway. Therefore, deep NMB, which requires a higher dose of muscle relaxants, may have more profound anti-inflammatory effects in the perioperative setting, compared to a standard block.

To date, studies have mainly focused on the effects of muscle relaxants on intraoperative surgical working conditions. The current EURO-RELAX trial was designed to investigate whether the application of a deep NMB over a standard NMB beneficially affects patient-centered outcomes, including intraoperative adverse events and postoperative complications. The trial is a multicenter randomized controlled, double blinded trial, stratified for surgical complexity and trial center. The trial is performed in 8 centers in Europe, encompassing 922 patients undergoing various types of elective laparoscopic or robotic surgery. We choose to include only procedures that are of moderate to high surgical complexity (i.e., BUPA class major, major plus, and complex major), as we believe that any beneficial effects of deep NMB will be more pronounced in these procedures.

The primary outcome of the trial is the incidence of intraoperative adverse events, rated on the ClassIntra® grade [11]. The ClassIntra® grade is the only prospectively validated classification to rate intraoperative adverse events. It has a high interrater reliability and includes both surgery- and anesthesia-related adverse events [11]. The primary outcome will be scored by the blinded surgical anesthesia team. Surgeons and anesthesiologists will be trained to score consistently. Any adverse event from the start of anesthesia, until the patient has left the OR, will be scored on the ClassIntra® grade. Adverse events that occur in the postoperative anesthesia care unit, and going forward, on the surgical ward and after discharge until 30 days after surgery, will be scored using the Clavien-Dindo scale. The Clavien-Dindo scale is a commonly used scale to grade adverse events and complications that are the result of surgical procedures. In addition to these outcomes, the patient-reported secondary outcomes (SF-36 and QoR-40) are analyzed to evaluate the patient perspective of the applied interventions.

### Trial limitations

First, the current trial was initiated in a time where inclusion may be restrained by local SARS-CoV-2 outbreaks. As such, inclusion may take longer than expected.

In this trial, both total intravenous and inhalational anesthetic agents may be used, the choice being at the discretion of the attending anesthesiologist, which is compliant with real-world practice. Although the benefit of deep NMB on surgical working conditions is less existent during inhalational anesthesia [31], a relationship with (safety-) outcomes and depth of NMB during either inhalational or intravenous anesthesia is not yet established. In addition, deep neuromuscular block may be difficult to reach and maintain in a subset of patients due to various reasons. However, our experience is that this only happens in a few patients. This will also reflect real-world practice and give a true indication of the treatment effect. Furthermore, scoring of the ClassIntra® grade may depend on the surgical anesthesia team who scores the outcome. All assessors will be trained prior to the trial, and a blinded adjudication committee will regularly discuss any scoring issues and ensure consistent scoring. Finally, the effect of deep NMB on outcomes may differ between various surgeries and depend on the type of anesthesia. These effects will be assessed in subgroup analyses.

In conclusion, the EURO-relax trial is the first multicenter randomized controlled trial that will assess the effect of deep neuromuscular block versus a standard neuromuscular block on major safety outcomes in laparoscopic surgery.

## Appendix

**Table 4:** EURO-Relax trial centers, address, and local contact

**France**	
Université De Lorraine Rue du Morvan, Nancy, 54000, France Contact: Thomas Fuchs-Buder	
**Italy**	
Istituto Nazionale Dei Tumori Via Venezian 1, Milano, 20133, Italy Contact: Franco Valenza	
**The Netherlands**	
Canisius Wilhelmina Ziekenhuis Weg door Jonkerbos 100, Nijmegen, 6532 SZ, The Netherlands Contact: Ivo Panhuizen	
Leiden University Medical Center Albinusdreef 2, Leiden, 2333 AL, The Netherlands Contact: Maarten Honing, MD	
Netherlands Cancer Institute Plesmanlaan 121, Amsterdam, 1066 CX, The Netherlands Contact: Suzanne Broens	
Noordwest Ziekenhuis Groep Wilhelminalaan 12, Alkmaar, 1815 JD, The Netherlands Contact: Margot H.J. Roozekrans, PhD, MD	
Radboud University Medical Center Geert Grooteplein-Zuid 10, Nijmegen, 6525GA, The Netherlands Contact: Michiel Warlé	
**Spain**	
Hospital Universitari I Politecnic La Fe Avinguda de Fernando Abril Martorell, 106, Valencia, 46026, Spain Contact: Oscar Díaz Cambronero	

## Abbreviations

ASA: American Society of Anesthesiologists
BAC: Blinded adjudication committee
BIS: Bispectral index
BUPA: British United Provident Association
CCI: Comprehensive Complication Index
CCMO: Central Committee on Research Involving Human Subjects
CRF: Case report form
DMC: Data monitoring committee
EDC: Electronic data capture
iAEs: Intraoperative adverse events
IAP: Intra-abdominal pressure
ICU: Intensive care unit
L-SRS: Leiden Surgical Rating Scale
MREC: Medical Research Ethics Committee
NMB: Neuromuscular block
NRS: Numeric rating scale
NSAIDs: Non-steroidal anti-inflammatory drugs
PACU: Post-anesthetic care unit
PTC: Post-tetanic count
(S)AE: (Serious) Adverse event
TOF: Train-of-four
